# Inguinal bladder hernia: A case report and literature review

**DOI:** 10.1016/j.ijscr.2019.04.040

**Published:** 2019-05-03

**Authors:** Adel Elkbuli, Raed Ismail Narvel, Mark McKenney, Dessy Boneva

**Affiliations:** a Department of Surgery, Kendall Regional Medical Center, Miami, FL, USA; b University of South Florida, Tampa, FL, USA

**Keywords:** CT, computed tomography, Inguinal hernia, Urinary bladder, Bladder hernia, Urinary leakage

## Abstract

**Introduction:**

Cases of inguinal bladder hernia are rare, with bladder involvement seen in 1–4% of inguinal hernias. The majority of cases are diagnosed intraoperatively, with only 7% of bladder hernias identified prior to surgery. Diagnosis may be challenging as patients are often asymptomatic or have nonspecific symptoms. Surgical repair is currently the standard treatment, and careful surgical planning is necessary to avoid complications including bladder injury.

**Presentation of case:**

A 58-year-old man presented to our Emergency Department with 2-day history of progressively worsening left lower quadrant pain, groin bulge, and dysuria. Physical exam revealed an irreducible left inguinal hernia associated with urinary urgency on attempted reduction. Plain CT was ordered and demonstrated inguinal hernia with bladder protrusion into the left scrotum. The patient underwent open surgical reduction and hernia repair and made a quick postoperative recovery without complications.

**Discussion:**

Inguinal bladder hernia most often presents in older, obese males and clinicians should have a high index of suspicion when assessing patients with inguinal hernia. Preoperative diagnosis based on history, physical, and radiologic imaging allow for careful surgical planning and prevention of severe complications including bladder injury and leakage.

**Conclusion:**

We present a case report of inguinal bladder hernia in a middle-aged man that presented as left lower quadrant pain, groin pain, and dysuria. Diagnosis was confirmed preoperatively with radiographic imaging. The hernia was surgically reduced and the defect repaired without complications.

## Highlights

• Inguinal bladder hernia is an unusual condition that requires a high index of clinical suspicion for diagnosis.
• This case demonstrates the importance of preoperative diagnosis in avoiding surgical complications including bladder injury.

## 1 Introduction

Inguinal bladder hernia, first described in the literature by Levine in 1951, is a rare condition despite the proximity of the bladder to the inguinal canal [1]. Bladder herniation is present in 1–4% of all inguinal hernias, though incidence may be as high as 10% in obese men over the age of 50 [2]. Risk factors include male gender, advanced age, chronic urinary obstruction, weak pelvic musculature and obesity [3]. Significantly, only 7% of inguinal bladder hernias are diagnosed prior to surgery, with the vast majority being diagnosed intraoperatively and 16% diagnosed postoperatively due to complications including bladder injury and leakage [4].

Diagnosis of inguinal bladder hernia may be challenging because the majority of patients are asymptomatic, in which case preoperative diagnosis depends on incidental discovery on radiography [5]. Patients that are symptomatic most often present with nonspecific symptoms such as inguinal swelling, dysuria, hematuria, and urinary urgency [6,7]. In advanced cases, patients must complete 2-stage urination in which they manually compress the scrotum after voiding for bladder emptying [5]. Severe urologic complications include urinary tract infections, obstructive uropathy, and even bladder infarctions that require subtotal cystectomy [5,8]. Associated pathologies include benign prostatic hyperplasia, hydronephrosis, vesicouretertic reflux, and scrotal abscesses [3].

Surgical repair of the hernia after bladder reduction is currently the standard treatment, and consists of intraoperative reduction or less commonly resection of bladder followed by herniorrhaphy [9]. If the diagnosis is known, catheterization is recommended prior to surgery [10]. Prompt recognition of inguinal bladder herniation and appropriate imaging prior to surgery can aid in planning for a modified surgical approach and lessen postoperative complications.

Here in, we describe the case of a 58-year-old obese man with left sided inguinal bladder herniation presenting as worsening abdominal pain, groin pain, and dysuria. This case has been reported in line with the SCARE criteria [11].

## 2 Case presentation

A 58-year-old obese male presented to our Emergency Department with 2-day history of progressively worsening left lower quadrant abdominal pain, urinary urgency, and 3/10 groin pain. The patient also reported dysuria. Comorbidities included hypertension and Class 1 obesity with body mass index of 32. He denied experiencing similar symptoms in the past and reported no other past medical history, no smoking history, or relevant family history.

**Fig. 1:**
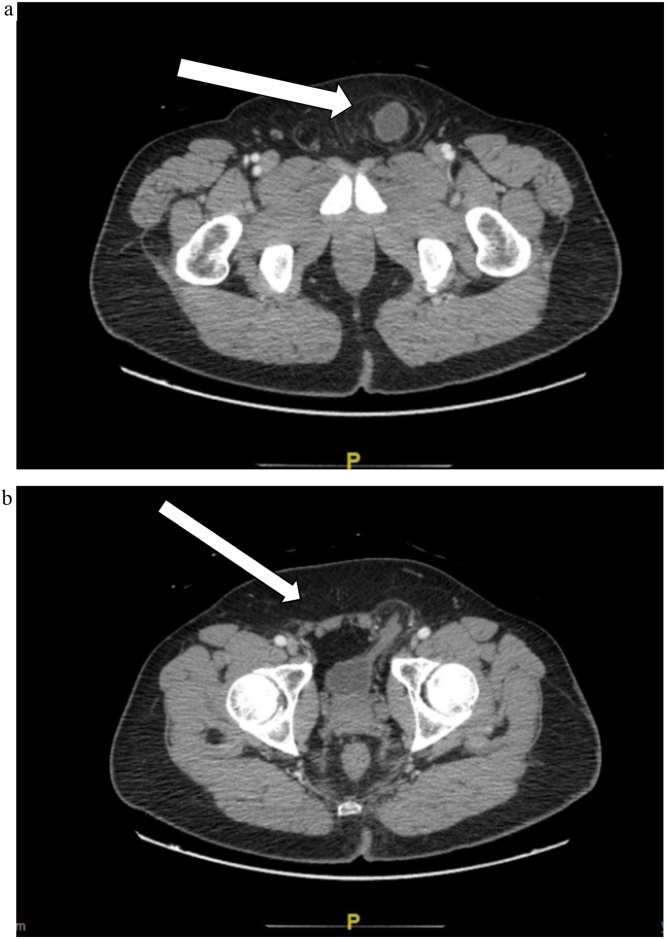
**A.** CT scan showing bladder herniated/protruded into the Left groin; axial view. **B.** CT scan of the pelvis shows a slice through the bladder intraperitoneally and a part of the bladder herniating through the Left inguinal hernia/groin. The urinary bladder is stretched into the left inguinal hernia. No bladder mass or calcifications seen/present.

Physical exam was significant for obvious 7 cm left inguinal hernia bulge with tenderness to palpation. On attempt to reduce the hernia, the patient reported urinary urgency. Laboratory studies and urinalysis were within normal limits. A computed tomography (CT) scan revealed left inguinal hernia containing a portion of the urinary bladder (Fig. 1A–C).

**Fig. 2:**
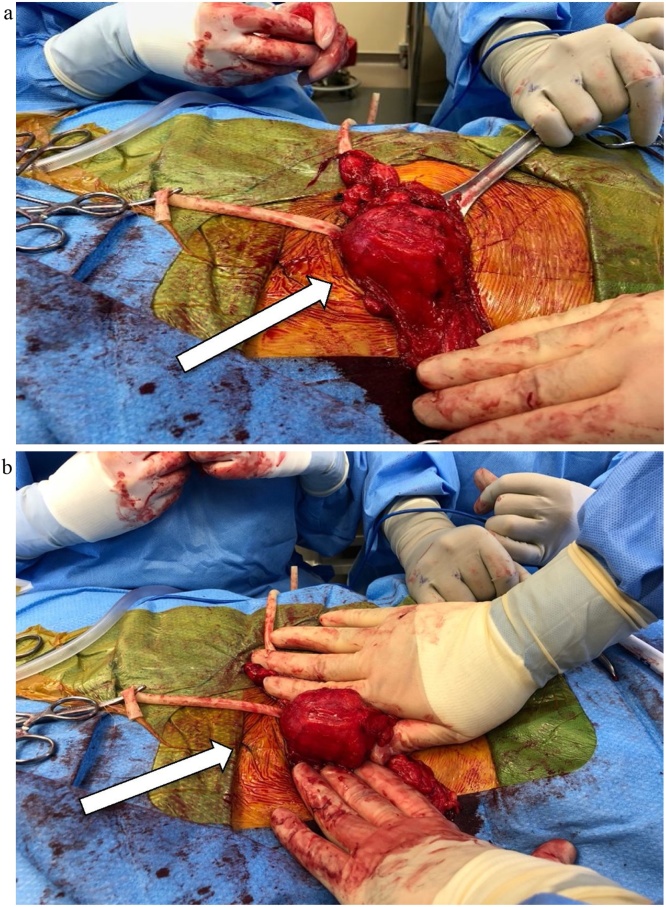
**A.** Left inguinal incision seen (patient’s head is to the right of the photo, the patient’s feet to the left of the photo); bladder starting to distend as the bladder catheter was being filled with sterile saline. Penrose drain seen around the inguinal cord structures. **B.** Distended bladder seen via the left inguinal incisions (head of the pt is to the right of the picture; feet of the pt to the left of the picture). The bladder is protruding through the inguinal/groin incision filled with sterile saline via the bladder catheter.

The patient was taken to the Operating Room the same day for planned open left inguinal hernia repair with mesh. Intra-operatively, a portion of the urinary bladder was found to be inside the inguinal hernia. The inguinal hernia sac was initially difficult to differentiate from the bladder due to its thickness (Fig. 2A, B). The bladder was subsequently distended with Normal Saline via the Foley catheter and a large bulge was observed in the inguinal canal, confirming the bladder herniation. The bladder was then reduced into the abdomen and the inguinal hernia and defect were fixed with a polypropylene mesh.

The patient had no intra-operative complications. On post-operative day (POD) 1, the patient appeared well with minimal groin pain. Incision was clean, dry, and with no drainage or signs of infection. Patient was able to ambulate and tolerate diet. His Foley catheter was removed and the patient was able to urinate without pain or difficulty. Patient was discharged to home on POD 2 with controlled pain, regular diet, and normalized white blood cell count. He was seen in clinic 2 weeks post-operatively free of pain and with no urinary voiding symptoms. Incision was clean, dry, and healed.

## 3 Discussion

Our patient was a 58-year-old man who presented with 2-day history of left lower quadrant abdominal pain, groin pain, and urinary urgency. Physical exam revealed an irreducible inguinal hernia with tenderness to palpation and feeling of urinary urgency on attempted reduction. CT revealed bladder involvement in the hernia. Preoperative diagnosis of inguinal bladder herniation allowed careful planning and a successful reduction of the bladder into the abdominal cavity and repair of the hernia defect with mesh.

The pathophysiology of an inguinal bladder herniation involves pulling of the bladder and a sheath of peritoneum that forms its sac through a weak point in the abdominal fascia [12]. Several factors may contribute to the development of inguinal bladder hernia including bladder outlet obstruction, weakness of pelvic musculature, decreased bladder tone, and obesity [10,12]. Risk factors include male gender, advanced age, and benign prostatic hypertrophy [9]. Patients are typically asymptomatic, however they may have nonspecific symptoms including urinary frequency, urgency, hematuria, and nocturia [7]. In rare cases of very severe bladder herniation, patients may describe two-stage micturition in which they feel the need to compress the scrotum in order to urinate [7]. Previous reports have described other rare cases including patients presenting with massive bladder herniation resulting in acute renal failure, bilateral hydronephrosis, inguinal bladder hernia masking bowel ischemia, and bladder hernia with ureteral obstruction years after kidney transplantation [3,6,7,13].

Careful history and physical examination is key in establishing a preliminary diagnosis. Ultrasonography, cystography, and CT have all been utilized to confirm the diagnosis [5]. Given the low number of bladder hernias that are diagnosed preoperatively, the clinician should have a high index of suspicion especially in older, obese, male populations in whom incidence has been reported as high as 10% of all inguinal hernia [2]. CT scan may be beneficial in obese males >50 with inguinal swelling and lower urinary tract symptoms, followed by cystoscopy to confirm diagnosis and rule out additional pathology of the bladder [12]. Voiding cystourethrography revealing a “dumbbell” or “dog-ear” shape of the bladder is the most sensitive test for diagnosis of inguinal bladder hernia, and can confirm a diagnosis without additional need for CT [14]. We recommend voiding cystourethrography in cases where the suspicion for inguinal bladder hernia is high, or in cases where initial imaging with ultrasound or CT is inconclusive.

Standard treatment of inguinal bladder hernia involves bladder reduction or on rare occasion partial resection followed by hernia repair, which historically was done with a midline laparotomy but more recently has been done with an open inguinal incision or with a laparoscopic approach [15]. Currently, bladder resection is recommended only in cases with bladder wall necrosis, true herniated bladder diverticulum, a tight hernia neck, or tumor in the herniated bladder [5]. Patients can also be treated conservatively with urethral catheterization to decompress and reduce the bladder [4]. In our case, normal saline was used to distend and better visualize the bladder and an open reduction and hernia repair with mesh was performed. Preoperative diagnosis allowed careful planning of the procedure, and the patient recovered well without complications.

## 4 Conclusion

Inguinal bladder hernia is an unusual condition that requires a high index of clinical suspicion for diagnosis. In this report, we demonstrate a case of inguinal bladder hernia presenting as worsening left lower quadrant pain, groin pain, and dysuria. Reduction of the hernia and bladder, followed by hernia repair with mesh was performed through an open approach. Bladder distension with normal saline was via a bladder catheter was utilized to better visualize the bladder and aid in reduction. The patient had no complications. This case demonstrates the importance of preoperative diagnosis in avoiding surgical complications including bladder injury.

## Conflict of interest

None.

## Funding

None.

## Ethical approval

This is a case report study. Informed written consent has been obtained and all identifying information is omitted. This work has been conducted in compliance with institutional ethical standards.

## Consent

Informed written consent has been obtained and all identifying information is omitted.

## Author contributions

Adel Elkbuli, Dessy Boneva, Raed Ismail Narvel, Mark McKenney – Conception of study, acquisition of data, analysis and interpretation of data.

Adel Elkbuli, Dessy Boneva, Raed Ismail Narvel – Drafting the article.

Dessy Boneva, Mark McKenney – Management of case.

Adel Elkbuli, Raed Ismail Narvel, Dessy Boneva, Mark McKenney – Critical revision of article and final approval of the version to be submitted.

## Registration of research studies

This is a case report study.

## Guarantor

Dessy Boneva.

Mark McKenney.

## Provenance and peer review

Not commissioned, externally peer-reviewed.
